# A combined association of obesity, alanine aminotransferase and creatinine with hyperuricemia in youth aged 13–20 years

**DOI:** 10.3389/fnut.2024.1326039

**Published:** 2024-06-20

**Authors:** Yang Niu, Yajie Zhang, Yan Sun, Jinye Sheng, Wenyi Lu, Ji Li, Xiaomeng Mao, Yi Feng, Xiuhua Shen

**Affiliations:** ^1^Department of Clinical Nutrition, Xinhua Hospital Affiliated to Shanghai Jiao Tong University School of Medicine, Shanghai, China; ^2^Department of Clinical Nutrition, College of Health Science and Technology, Shanghai Jiao Tong University School of Medicine, Shanghai, China; ^3^Shanghai Institute for Pediatric Research, Shanghai, China; ^4^Shanghai Key Laboratory of Pediatric Gastroenterology and Nutrition, Shanghai, China; ^5^Department of Clinical Nutrition, Shanghai Chest Hospital, School of Medicine, Shanghai Jiao Tong University, Shanghai, China

**Keywords:** hyperuricemia, youth, body mass index, alanine aminotransferase, creatinine

## Abstract

**Background:**

Despite extensive research on hyperuricemia (HUA) in adults, there remains a dearth of studies examining this condition in youth. Consequently, our objective was to investigate the prevalence of HUA among youth in the United States, as well as identify the corresponding risk factors.

**Methods:**

This study employed a nationally representative subsample of 1,051 youth aged 13–20 from the US National Health and Nutrition Examination Survey (NHANES) conducted between January 2017 and March 2020. Univariate and multivariate techniques were utilized to examine the association between HUA and obesity, dietary nutrients, liver and kidney function, glucose and lipid metabolism, inflammation, and other indicators in the adolescent population.

**Results:**

The study encompassed a cohort of 1,051 youth aged 13–20 years, comprising 538 boys and 513 girls. The overall prevalence of HUA was found to be 7% (74 out of 1,051). Univariate analysis revealed that the HUA group exhibited greater age, body mass index (BMI), waist circumference (WC), hip circumference (HC), and waist-to-hip ratio (WHR). Additionally, the prevalence of obesity was significantly higher in the HUA group compared to the non- HUA group (all *p* < 0.05). Regarding biochemical indicators, the levels of urea nitrogen, creatinine (Cr), alanine aminotransferase (ALT), glutamic oxalic aminotransferase (AST), gamma-glutamyl transferase (GGT), total cholesterol (TC), triglyceride (TG), and HS C reactive protein (Hs CRP) were found to be significantly higher in the HUA group compared to the non-HUA group (all *p* < 0.05). Further analysis using binary logistics regression showed that BMI (*p* = 0.024, OR1.158, 95%CI1.019–1.316), ALT (*p* = 0.020, OR1.032, 95%CI1.005–1.059), and Cr (*p* = 0.016, OR1.028, 95%CI1.005–1.051) were identified as risk factors for HUA, after controlling for age, gender, BMI, WC, HC, WHR, ALT, AST, GGT, TG, TC, Cr, Hs CRP, and other indicators. Interestingly, neither univariate nor multivariate analysis found any association between dietary nutrients and the risk of HUA (all *p* > 0.05).

**Conclusion:**

High BMI remains a major risk factor for HUA in US youth aged 13–20 years, and ALT and Cr levels should be closely monitored along with serum uric acid.

## Introduction

Over the last few decades, there has been a significant rise in the prevalence of hyperuricemia (HUA), making it a major global public health concern (1–3). HUA has emerged as a common metabolic disorder, alongside diabetes, hyperlipidemia, and hypertension. Among adults, HUA rates have been reported to range from 11.3 to 47% in the United States, 11.9 to 25.0% in Europe, and 26.8% in Japan (4). Regrettably, the increasing occurrence of HUA in children is often overlooked, as it is commonly perceived as a condition affecting only adults.

However, as the occurrence of HUA continues to rise, it is becoming more common among younger individuals. According to data from the National Health and Nutrition Examination Survey, a nationally representative sample in the United States, the prevalence of HUA among 1,399 adolescents aged 12–19 years between 2013 and 2016 was 16.56% (5). Furthermore, the prevalence of HUA increased as age advanced, with rates of 3.7, 9.8, 15.8, 35.5, and 31.7% for age groups 3–5, 6–8, 9–11, 12–15, and 16–19 years, respectively (*p* < 0.001). Additionally, the prevalence of HUA showed a faster increase among children of different weight categories, including non-overweight, overweight, obese, and extremely obese, with rates reaching 18.2, 37.6, 50.6, and 64.5%, respectively (*p* < 0.001) (6).

Although the effect of elevated uric acid levels on the pathogenesis of metabolic syndrome is still not fully understood (7, 8), HUA has been found to be closely associated with the risk of gout, diabetes (9, 10), fatty liver (11), hypertension (12), cardiovascular disease (13, 14), and chronic kidney disease (15). It is imperative that we should place greater emphasis on HUA in children and actively assess the associated risk factors. While there is evidence supporting the association between body mass index (BMI), waist circumference (WC), hip circumference (HC), intake of various dietary nutrients, liver function, glucose and lipid metabolism, inflammation, and other factors, their comprehensive consideration in current studies on youth population with HUA is limited. As a result, this study aims to conduct a survey among youth, incorporating common biochemical and anthropometric parameters, and identify potential risk factors associated with HUA in this population. The findings of this study will serve as a scientific foundation for the prevention and management of HUA in youth.

## Materials and methods

### Study design and participants

We examined data from the US National Health and Nutrition Examination Survey (NHANES) conducted between 2017 and March 2020. After excluding incomplete and missing data, a total of 1,051 youth aged 13–20 years (16) were included in the study. The serum uric acid (SUA) levels of the participants were compared to the reference values provided by Mayo Clinic Laboratories, which vary based on sex and age (17). For boys, the reference values are as follows: 13 years: 3.4–6.9 mg/dL, 14 years: 3.7–7.4 mg/dL, 15 years: 4.0–7.8 mg/dL, and ≥ 16 years: 3.7–8.0 mg/dL. For girls, the reference values are ≥13 years: 2.7–6.1 mg/dL. For participants above 18 years of age, HUA was defined as 7.0 mg/dL for males and 6.0 mg/dL for females (18). Based on these reference values, the participants were categorized into two groups: HUA group and non-HUA group.

### Data collections and measurement

#### Demographic characteristics

Demographic factors encompass various attributes such as age, gender, height, weight, waist circumference (WC), hip circumference (HC), and ethnicity, which includes categories such as Mexican American, other Hispanic, Non-Hispanic White, Non-Hispanic Black, Non-Hispanic Asian, and other race - including Multi-Racial. Body Mass Index (BMI) and waist-to-hip ratio (WHR) can be determined using the following formulas: BMI = weight (in kilograms) divided by height (in square meters); WHR = waist circumference (in centimeters) divided by hip circumference (in centimeters). Based on BMI, individuals can be categorized as having low weight, normal weight, overweight, or obese (19).

#### Laboratory data

Laboratory markers commonly used to assess liver function, kidney function, and metabolic health include alanine aminotransferase (ALT), aspartate aminotransferase (AST), gamma-glutamyl transferase (GGT), blood urea nitrogen (BUN), creatinine (Cr), SUA, blood glucose (Glu), hemoglobin A1c (HbA1c), triglyceride (TG), total cholesterol (TC), and high-sensitivity C-reactive protein (Hs CRP). Following the guidelines provided by the North American Society of Pediatric Gastroenterology, Hepatology and Nutrition (Naspugen), elevated ALT levels are defined as reaching 22 IU/L for females and 26 IU/L for males (20).

#### Dietary assessment

The study collected data on the average intake of dietary nutrients by conducting two 24-h dietary recall interviews. In cases where only one recall was completed by participants, the data from that recall was used. The main dietary nutrients included in this study were energy, protein, carbohydrate, fat, and the percentage of these three major nutrients in total energy. The calculations were done as follows: the percentage of protein in total energy = protein *4/ energy; the percentage of carbohydrate in total energy = carbohydrate *4/ energy; the percentage of fat in total energy = fat *9/ energy. Additionally, the study also considered other nutrients such as sugar, dietary fiber, saturated fatty acids, monounsaturated fatty acids, polyunsaturated fatty acids, cholesterol, niacin, folic acid, vitamin C, moisture, and total plain water. The daily total water intake was calculated as the sum of moisture and total plain water.

### Statistical analysis

The data analysis was conducted using the SPSS version 25 software. Descriptive statistics were used to summarize the characteristics of participants. For continuous variables, the median (p25, p75) was used to represent the data, while percentages (%) were used for categorical variables.

The study participants were divided into two groups, namely the HUA group and the non-HUA group, based on their SUA levels. To examine the correlation between HUA and various factors such as obesity, diet, liver and kidney function, glucose and lipid metabolism, inflammation, and other indicators, several statistical tests were employed. These tests included the independent sample t-test, chi-square test, and spearman correlation test. In addition, a binary logistic regression analysis was conducted to assess the influence of different variables on the risk of developing HUA, with uric acid being the dependent variable. Model 1 was controlled for BMI, WC, HC, WHR, ALT, AST, GGT, TG, TC, Cr, and Hs CRP. Model 2, on the other hand, included the control variables from Model 1 as well as age and gender. All *p*-values were calculated using a bilateral test, and a significance level of *p* < 0.05 was adopted for each test.

## Results

### Basic characteristics of participants

This study enrolled a total of 1,051 youth between the ages of 13 and 20, comprising 538 boys and 513 girls. Among the 1,051 participants, 7% (74/1051) were found to have HUA. The HUA group exhibited significantly higher age, height, weight, BMI, WC, HC, and WHR compared to the non-HUA group (all *p* < 0.05). Additionally, the HUA group had a significantly higher prevalence of obesity (*p* < 0.05).

In Table 1, the biochemical indexes, including BUN, Cr, SUA, ALT, AST, GGT, TC, TG, and Hs CRP, were found to be higher in the HUA group compared to the non-HUA group (all *p* < 0.05). Specifically, ALT levels in the HUA group were significantly higher than in the non-HUA group (45.9% Vs. 12.3%) (all *p* < 0.05, Table 1). However, there were no significant differences in Glu and HBA1c levels between the HUA group and the non-HUA group (both *p* > 0.05).

**Table 1:** Basic characteristics of participants in HUA group and non-HUA group.

	Non-HUA group (*n* = 977)	HUA group (*n* = 74)	*p* value
*n*, %	977,93	74,7	
Sex, boys/girls	485 / 492	53 / 21	<0.001
Age, y	16 (14, 18)	17.5 (14, 19)	0.047
Race, *n* (%)			
Mexican American	153 (15.7)	19 (25.7)	0.166
Other Hispanic	87 (8.9)	3 (4)	
Non-Hispanic White	306 (31.3)	18 (24.3)	
Non-Hispanic Black	246 (25.2)	17 (23)	
Non-Hispanic Asian	102 (10.4)	9 (12.2)	
Other Race - Including Multi-Racial	83 (8.5)	8 (10.8)	
Height, cm	165.9 (159.5, 173.7)	169.5 (163.4, 177.1)	0.004
Weight, kg	64.9 (54.8, 79.7)	90.9 (75.1, 109.1)	<0.001
BMI, kg/㎡	23.3 (20.2, 27.6)	32.2 (25.2, 38.5)	<0.001
Weight status, *n* (%)			
Low weight	37 (3.8)	1 (1.3)	<0.001
Normal weight	557 (57)	19 (25.7)	
Overweight	164 (16.8)	8 (10.8)	
Obese	219 (22.4)	46 (62.2)	
WC, cm	79.3 (71.6, 91)	101.4 (86.7, 114)	<0.001
HC, cm	101.4 (86.7, 109.5)	108.5 (100.3, 120.1)	<0.001
WHR	0.8 (0.7, 0.9)	0.9 (0.8, 1.0)	<0.001
BUN, mmol/L	3.9 (3.2, 5)	4.2 (3.5, 5)	0.436
Cr, mmol/L	63.6 (54.8, 74.2)	69.8 (64.3, 81.5)	<0.001
SUA, mg/dL	4.9 (4, 5.7)	7.4 (7, 8.1)	<0.001
ALT, U/L	13 (10, 18)	23 (14.7, 36.2)	<0.001
AST, U/L	18 (15, 21)	21 (17.7, 27)	<0.001
GGT, U/L	13 (10, 18)	19.5 (15, 30.5)	<0.001
Elevated ALT (≥ 26 U/L in boys, ≥ 20 U/L in girls), *n* (%)	120 (12.3)	34 (45.9)	<0.001
TC, mmol/L	3.9 (3.5, 4.4)	4.1 (3.6, 4.8)	0.012
TG, mmol/L	0.8 (0.6, 1.1)	1.1 (0.8, 1.8)	<0.001
Glu, mmol/L	4.8 (4.6, 5.1)	4.9 (4.6, 5.1)	0.256
HbA1c, %	5.3 (5.1, 5.5)	5.3 (5.1, 5.5)	0.479
Hs CRP, mg/L	0.6 (0.3, 1.6)	1.5 (0.6, 4)	<0.001

WC, waist circumference; HC, hip circumference; WHR, waist-to-hip ratio; BUN, blood urea nitrogen; Cr, creatinine; SUA, serum uric acid; ALT, alanine aminotransferase; AST, aspartate aminotransferase; GGT, gamma-glutamyl transferase; TC, gamma-glutamyl transferase; TG, triglyceride; Glu, blood glucose; HbA1c, hemoglobin A1c; Hs CRP, high-sensitivity C-reactive protein.

### Dietary intake characteristics of participants

According to the findings presented in Table 2, the dietary nutrient intake of the HUA group showed lower levels of energy, carbohydrate, fat, protein percentage, dietary fiber, and other nutrients compared to the non-HUA group (all *p* > *0.05*). Conversely, the HUA group demonstrated higher percentages of protein, sugar, carbohydrate, fat, saturated fatty acid, monounsaturated fatty acid, polyunsaturated fatty acid, niacin, folic acid, and vitamin C compared to the non-HUA group (all *p* > 0.05).

**Table 2:** Characteristics of dietary intake in HUA group and non-HUA group.

	Non-HUA group (*n* = 977)	HUA group (*n* = 74)	*p* value
Energy, kcal/d	1875 (1,483, 2,401)	1817.3 (1,431, 2304.6)	0.622
Protein, g/d	66.9 (50.6, 89.4)	70.2 (48.3, 86.3)	0.574
Carbohydrate, g/d	233.1 (182.1, 292.5)	225.3 (178.2, 300.7)	0.948
Sugar, g/d	92.9 (66.3, 130.3)	96 (61.8, 149.3)	0.592
Fat, g/d	76.8 (58.3, 98.1)	71.9 (54.5, 96.4)	0.476
Percentage of protein energy,%	14.2 (11.9, 16.7)	13.8 (11.3, 16.8)	0.574
Percentage of carbohydrate energy,%	49.4 (44, 54.6)	50.2 (43.4, 57.9)	0.948
Percentage of fat energy,%	32.2 (28.1, 36.5)	34.6 (31.5, 42)	0.476
Saturated fatty acids, g/d	21.9 (17.6, 30.2)	25.8 (18.3, 34.1)	0.107
Monounsaturated fatty acids, g/d	24.5 (18.9, 33.1)	25.3 (17.2, 32.4)	0.709
Polyunsaturated fatty acids, g/d	16.7 (12.3, 23.8)	17.8 (13.1, 24)	0.691
Fiber, g/d	12.8 (9.5, 17.4)	12.1 (8.5, 16)	0.267
Niacin, mg	21.1 (16.1, 28.4)	21.9 (15.8, 28.9)	0.992
Folate, mg	309.5 (223.2, 433.5)	312.7 (224.2, 420.5)	0.670
Vitamin C, mg	44.6 (19.1, 86.8)	45 (24, 86.5)	0.749

### Univariate analysis of factors associated with SUA

Table 3 presents the results of the spearman correlation test analysis. It was found that SUA exhibited positive correlations with age, height, weight, BMI, WC, HC, WHR, BUN, Cr, ALT, AST, GGT, TG, Glu, energy, protein, carbohydrate, sugar, fat, protein percentage, saturated fatty acids, monounsaturated fatty acids, polyunsaturated fatty acids, niacin, folic acid, and other indicators (all *p* < 0.05). On the other hand, HUA showed positive correlations with TC, dietary fiber, vitamin C, and other indexes, although these correlations did not reach statistical significance (all *p* > 0.05).

**Table 3:** Correlation analysis of indicators with SUA in the total sample.

	*r*	*p* value
Age	0.063	0.041
Height	0.435	<0.001
Weight	0.421	<0.001
BMI	0.277	<0.001
WC	0.333	<0.001
HC	0.263	<0.001
WHR	0.334	<0.001
BUN	0.228	<0.001
Cr	0.436	<0.001
SUA	1.000	<0.001
ALT	0.421	<0.001
AST	0.335	<0.001
GGT	0.389	<0.001
TC	0.015	0.635
TG	0.191	<0.001
Glu	0.101	0.001
HbA1c	0.028	0.370
Energy	0.148	<0.001
Protein	0.198	<0.001
Carbohydrate	0.126	<0.001
Sugar	0.090	0.004
Fat	0.097	0.002
Percentage of protein energy	0.104	0.001
Percentage of carbohydrate energy	−0.037	0.237
Percentage of fat energy	−0.049	0.112
Saturated fatty acids	0.071	0.022
Monounsaturated fatty acids	0.102	0.001
Polyunsaturated fatty acids	0.066	0.032
Fiber	0.058	0.062
Niacin	0.189	<0.001
Folate	0.104	0.001
Vitamin C	0.005	0.879

WC, waist circumference; HC, hip circumference; WHR, waist-to-hip ratio; BUN, blood urea nitrogen; Cr, creatinine; SUA, serum uric acid; ALT, alanine aminotransferase; AST, aspartate aminotransferase; GGT, gamma-glutamyl transferase; TC, gamma-glutamyl transferase; TG, triglyceride; Glu, blood glucose; HbA1c, hemoglobin A1c.

### Binary logistic regression of indicators with the risk of HUA

To investigate the impact of various indicators on the occurrence of HUA in youth, we conducted binary logistic regression analysis using the data from Table 4. After controlling for BMI, WC, HC, WHR, ALT, AST, GGT, TG, TC, Cr, and Hs CRP, we identified that BMI (*p* = 0.029, OR 1.149, 95% CI 1.014–1.301), ALT (*p* = 0.014, OR 1.032, 95% CI 1.006–1.059), and Cr (*p* = 0.001, OR 1.031, 95% CI 1.013–1.050) were risk factors for HUA. Additionally, when further adjusting for age and sex, the results remained consistent, with BMI (*p* = 0.024, OR 1.158, 95% CI 1.019–1.316), ALT (*p* = 0.020, OR 1.032, 95% CI 1.005–1.059), and Cr (*p* = 0.016, OR 1.028, 95% CI 1.005–1.051) being identified as risk factors for HUA.

**Table 4:** Logistic regression of indicators with the risk of HUA in the total sample.

	Model 1		Model 2	
	*p* value	OR (95% CI)	*p* value	OR (95% CI)
Age	–	–	0.593	0.963 (0.839,1.105)
Sex	–	–	0.398	1.382 (0.652,2.929)
BMI	0.029	1.149 (1.014,1.301)	0.024	1.158 (1.019,1.316)
ALT	0.014	1.032 (1.006,1.059)	0.020	1.032 (1.005,1.059)
Cr	0.001	1.031 (1.013,1.050)	0.016	1.028 (1.005,1.051)

Model 1: adjusted for BMI, WC, HC, WHR, ALT, AST, GGT, TG, TC, Cr, HS CRP; Model 2: Model 1+ age and sex. BMI, body mass index; ALT, alanine aminotransferase; Cr, creatinine.

## Discussion

Among the 1,051 youth aged 13–20 years in the United States who participated in this study, the detection rate of HUA was found to be 7%. The results revealed that the group with HUA had a higher BMI, prevalence of obesity, ALT, AST, GGT, and Cr. Furthermore, BMI, ALT, and Cr were identified as independent risk factors for the development of HUA. However, no significant associations were observed between glycolipid metabolism indexes and dietary factors in relation to the occurrence of HUA, warranting further investigation.

In a Korean study involving 1,256 children aged 12–18 years, the researchers discovered that the rate of HUA detection was approximately 9.1% (21), which aligns closely with the findings of our study. In the study of adolescents with different body weight, it was found that the detection rates of HUA in 12–15 and 16–19 year-olds in China were 35.5 and 31.7% (6), higher than the 7% detection rate in this study and 8.6–12.1% detection rate in previous study (22). This difference may be attributed to the high proportion of overweight and obese individuals in the study conducted by Rao et al. (6), or the adoption of different diagnostic criteria in different studies. Multivariate logistic regression analysis showed that even after controlling for age, gender, and other confounding factors, higher BMI remained a risk factor for HUA, which is consistent with previous research findings (21, 23).

Obesity is commonly defined as the abnormal or excessive accumulation of fat, and it has been widely recognized to have negative impacts on an individual’s health (24). Scholars both domestically and internationally have shown a growing interest in understanding the relationship between obesity and the occurrence and progression of HUA (6, 25, 26). According to previous studies conducted in children and adolescents aged 12–19 years, it was found that those in the HUA group had significantly higher BMI compared to those in the normal uric acid group (5, 27). In our previous study, we discovered that the prevalence of HUA in obese children was alarmingly high, reaching 47.9% (130 out of 271) (26). Additionally, the present study revealed a significant increase in BMI among individuals with HUA, particularly those who were classified as obese (62.2%).

In addition to BMI, which is known to increase the risk of HUA, other obesity indicators such as WC, HC, WHR, and fat accumulation also contribute to the development of HUA (28, 29). However, when comparing these indicators, BMI has a greater impact on HUA than the others (25). Interestingly, our study revealed that only BMI was identified as a significant risk factor for HUA after conducting multivariate logistic regression analysis. Conversely, WC, HC, and WHR did not show any significant association with HUA.

Obesity in individuals with HUA is believed to be influenced by several factors (27, 30, 31). Firstly, excessive accumulation of adipose tissue in obese individuals may disrupt the regulation of adipocytokines and trigger the release of inflammatory cytokines, leading to an increased production of uric acid. Secondly, HUA may be associated with the processes of lipogenesis and/or lipolysis. In the case of lipogenesis, the synthesis of fatty acids in fat cells results in the accumulation of triglycerides and an elevated purine synthesis, which subsequently leads to elevated SUA levels. Furthermore, this study found higher levels of TG and TC in the HUA group compared to the non-HUA group, which was consistent with the results of Jung Hyun Lee’s study (21). However, the results of the multivariate analysis did not indicate that the lipid metabolism index was a significant risk factor for HUA. This may be due to the fact that lipid metabolism only plays a connecting role in the development of HUA, rather than being a direct risk factor. Previous research has demonstrated that the excessive build-up of visceral fat triggers the release of a high amount of plasma-free fatty acids into the hepatic portal vein and liver. This, in turn, stimulates the synthesis of TG and subsequently leads to an increase in the production of uric acid by activating the uric acid synthesis pathway (32).

A study in the United States involving 5,370 adults found the prevalence of non-alcoholic fatty liver disease with increasing levels of SUA (33), and patients with HUA were more likely to have elevated levels of ALT and AST (33, 34). The higher levels of liver function in the HUA group may be attributed to the higher proportion of overweight and obese individuals in this population, which was found to be as high as 73% in this study. Previous studies have found that obesity plays a central role in the accumulation of fat in the liver and increases the liver’s vulnerability to injury (35–37). Additionally, it is important to note that the coexistence of HUA and obesity has a significant synergistic effect on hepatic steatosis (38). Impairment of liver cell function also leads to disorders in purine metabolism. Researches have demonstrated that both adults and children and adolescents with fatty liver disease have higher levels of SUA, which serves as an independent risk factor (39, 40). In essence, HUA and liver function damage are closely associated and may mutually influence each other, warranting further investigation into this phenomenon.

In our study, we made an interesting observation that the levels of BUN and Cr were found to be significantly higher in the HUA group compared to the non-HUA group. This finding is particularly noteworthy as it suggests that Cr could be an independent risk factor for HUA. However, it remains uncertain whether the higher levels of BUN and Cr in the HUA group are a result of impaired renal excretion, increased renal load, or other kidney diseases that lead to reduced elimination of uric acid. In previous study, the decreased estimated glomerular filtration rate (eGFR) was related to high HUA incidence in the logistic regression analysis (27). Further research in this area is needed to shed light on the mechanisms at play and determine the precise relationship between renal function and HUA. Such insights could have significant implications for the diagnosis and management of HUA in the future.

In terms of diet, our univariate results found that dietary energy, protein, carbohydrate, sugar and fat were positively correlated with SUA. Surprisingly, after binary logistic regression analysis, this study did not find any evidence to support the commonly held belief that different dietary nutrients play a significant role in the development of HUA in youth, contradicting previous research (41, 42). It may be considered that the main influencing factors of HUA are different in different age groups. Further research is necessary to investigate this phenomenon and provide more insights into the relationship between diet and HUA in youth.

Certainly, there are both shortcomings and advantages to consider in this study. Firstly, it is important to note that the study only collected a limited amount of dietary data from participants in NHANES 2017–2020. This may have restricted the accuracy and comprehensiveness of the findings. Secondly, the study was unable to establish a causal relationship between HUA and the indicators being measured.

Despite these limitations, our study has several notable strengths. It utilized data from the largest nationally representative sample of adolescents, allowing for findings to be generalized to a broader population. Additionally, this study defined reference ranges of SUA for HUA across various age groups and by gender. Furthermore, this study conducted a comprehensive examination of the relationship between HUA and various obesity indicators, dietary factors, liver and kidney function, glucose and lipid metabolism, as well as inflammation indicators. Importantly, this is the first study to assess these relationships specifically in youth.

In conclusion, obesity remains a significant risk factor for HUA in the United States youth aged 13–20 years. It is important to closely monitor ALT, Cr, and SUA levels in this population. Most concerning, in light of current research findings, we must work to improve the lifestyles of adolescents to prevent an epidemic of lifestyle-related diseases.

## Data availability statement

The original contributions presented in the study are included in the article/supplementary material, further inquiries can be directed to the corresponding authors.

## Ethics statement

The studies involving humans were approved by The institutional review board approved the NHANES protocol of the Centers for Disease Control and Prevention (CDC), and each participant provided written informed consent. The studies were conducted in accordance with the local legislation and institutional requirements. Written informed consent for participation in this study was provided by the participants' legal guardians/next of kin.

## Author contributions

YN: Conceptualization, Data curation, Formal analysis, Investigation, Methodology, Project administration, Validation, Writing – original draft, Writing – review & editing. YZ: Conceptualization, Data curation, Investigation, Methodology, Writing – original draft, Writing – review & editing. YS: Data curation, Investigation, Methodology, Writing – review & editing. JS: Data curation, Methodology, Writing – review & editing. WL: Data curation, Methodology, Supervision, Writing – review & editing. JL: Writing – review & editing. XM: Data curation, Methodology, Writing – review & editing. YF: Data curation, Methodology, Supervision, Writing – original draft, Writing – review & editing. XS: Data curation, Methodology, Supervision, Writing – original draft, Writing – review & editing.
